# Effects of Angipars on oxidative inflammatory indices in a murine model of periodontitis

**Published:** 2010

**Authors:** M. Mousavi-Jazi, H. Aslroosta, A.R. Moayer, M. Baeeri, M. Abdollahi

**Affiliations:** 1 Department of Periodontology, School of Dentistry and Dental Research Center, Tehran University of Medical Sciences; 2 Department of Periodontology, School of Dentistry, Islamic Azad University; 3 Faculty of Pharmacy, and Pharmaceutical Science Research Center, Tehran University of Medical Sciences, Tehran, Iran

**Keywords:** Periodontitis, Oxidative stress, Natural medicine

## Abstract

**Background and the purpose of the study:**

There are strong evidences linking overproduction of reactive oxygen species and periodontal diseases. The aim of this study was to evaluate efficacy of Angipars a natural potent anti oxidative agent on markers of the oxidative damages and periodontal inflammation in the rat.

**Methods:**

Periodontitis was induced by single injection of lipopolysaccharide (LPS) from *E. coli* (10 µg/µl saline) into rat mandibular gingiva. After 10 days, animals in the test group received Angipars (2.1 mg/kg) by gavage once a day and those of control group received same amount of vehicle. The amount of interleukin (IL)-1β, lipid peroxidation (LPO), and 8-hydroxydeoxyguanosine (8-OHdG) were measured in gingival biopsy samples and the degree of apical migration of junctional epithelium (JE), alveolar bone resorption, and the number of polymorphonuclears (PMN) were evaluated by histological analysis of block samples of the left mandibular first molars.

**Results:**

Periodontitis group showed a significant increase in periodontal IL-1β, LPO, 8-OHdG, apical migration of JE, alveolar bone resorption and number of PMNs. Angipars treatment resulted in a significant decrease in gingival IL-1β, LPO, 8-OHdG and the apical migration of JE; however, the reduction of alveolar bone resorption was not significant. The number of PMN increased significantly after treatment with Angipars. While intake of vehicle resulted in a significant decrease in gingival IL-1β and LPO, the reduction of 8-OHdG, apical migration of JE, and alveolar bone resorption were not significant. Interestingly, PMNs were increased in groups received Angipars or the vehicle.

**Conclusion:**

From the results of this study, it seems that Angipars is beneficial in periodontitis by reduction of inflammatory and oxidative damage. Unexpected increase of PMN count by Angipars strengthens the hypothesis that chronic inflammatory disorders like periodontitis may need more time to get best advantage of anti oxidative drugs like Angipars. Regarding role of microbes in pathogenesis of periodontitis, further studies should be focused on antimicrobial effects of Angipars.

## INTRODUTION

Periodontal diseases including gingivitis and periodontitis are the most common chronic inflammatory diseases among populations all over the world (1). The most important etiological factor for periodontal diseases is bacteria like *Porphyromonas gingivalis*, *Actinobacillus actinomycetem commitans*, and *Tanerella forsythia* (2). In patients with chronic periodontitis, an increase in oxidant products in serum, saliva and gingival crevicular fluid (GCF) (3) and a decrease in antioxidant capacity of GCF (4) have been reported. Also, inflammatory cytokines such as interleukin (IL)-1β (5), IL-6 (6), cyclic nucleotides (7), and nitric oxide (NO) (8) increased in periodontitis caused pathologically or upon adverse drug reactions (9).

Angipars (Semelil) is a registered drug derived from a plant named *Melilotus officinalis* under electromagnetic processes which contains compounds such as 7-hydroxycoumarin and flavonoidsused and is available as a suspension in 9.2% ethanol. Angipars has passed safety and clinical trials processes and has been registered as a drug of choice for management of human diabetic foot (10–13). Coumarin which is the main component of this drug is known for its anti-inflammatory and antioxidant activity, and ability to suppress superoxide and NO production in leukocytes, thereby, to reduce phagocyte activity (14).

The aim of this study was to evaluate the efficacy of Angipars on experimental periodontitis by measurement of periodontal oxidative inflammatory and histological parameters.

## MATERIAL AND METHODS

Thirty six male Wistar rats (12 weeks of age) were housed in an air conditioned room (23–25°C) with a 12 hrs dark and 12 hrs light cycle. The experimental protocol was approved by the institute review board. All chemicals were obtained from Sigma-Aldrich Chemie (GmbhMunich, Germany) unless otherwise stated. LPO and IL-1β ELISA kits were purchased from Bendermed system (Austria) and DNA damage ELISA kit was purchased from Cayman (USA). Angipars was obtained from Parsrus Research Group (Tehran, Iran).

### 

#### Induction of experimental periodontitis and treatments

The rats were divided into four groups (9 in each group). One group received no treatment for the whole study and was assigned as normal group. Periodontitis was induced in three test groups by injection of 10 µg of lipopolysaccharide (LPS) from *E. coli* in 1 µl saline into rat mandibular gingival at the distiobaccal aspect of the first molar (15). After 10 days (the time required for initiation of periodontitis), one of these groups (periodontitis group), was sacrificed under general anesthesia by overdose of ketamine. One of the remaining two groups (Angipars group) was treated with Angipars (2.1 mg/kg) by gavage once a day for 2 weeks. The other group assigned as vehicle group and received ethanol 9.2%. After 2 weeks, the animals were sacrificed by overdose of ketamine and then the left mandibular site was resected from each rat and fixed in formaldehyde (10%) in phosphate buffer (pH of 7.4; 0.1 mol/l) for 1 day. Gingival biopsy samples of the right mandibulare first molar were immediately frozen and kept at −80°C and then homogenized at the appropriate time.

The formaldehyde-fixed samples were further processed and stained with hematoxylin and Eosin and then were examined microscopically by a pathologist to assess the distance between the cementoenamel junction and the alveolar crest, the distance between the cementoenamel junction (CEJ) and the most coronal portion of the junctional epithelium (JE), and the polymorphonuclear leukocytes (PMN) in the connective tissue adjacent the JE.

The concentration of IL-1β,8-hydroxydeoxyguanosine (8-OHdG; measure of DNA damage), and cellular lipid peroxidation (LPO) were measured according to manufacturer's instruction.

#### Statistical analyses

All values in the figures and text are presented as mean±SE. Data were first examined for normality by the Kolmogorov-Smirnov test and if data did not achieve normality, analysis was performed using non-parametric methods. The values of apical migration of junctional epithelium and PMN count did not achieve normality. Univariate analysis of variance, followed by Tukey's test were used to compare the values of 8-OHdG, LPO and alveolar bone resorption. Levene test was used to compare IL-1β values. The difference in values of attachment level and PMN count between groups was analyzed using the Kruskal-Wallis test.

## RESULTS AND DISCUSSION

Animals of the periodontitis group showed a significant increase in gingival IL-1β, DNA damage and LPO levels as compared with normal group. Meanwhile, Angipars significantly reduced LPS induced elevation of gingival IL-1β, DNA damage, and LPO. Interestingly, gingival IL-1β and LPO in the vehicle group was significantly reduced when compared with periodontitis group. Of course, DNA damages in vehicle group was not different from periodontitis group but was significantly higher than that of normal group. Consistent with this finding, it has been reported that gingival LPO in periodontitis is elevated through production of superoxide anion during interaction between bacterial LPS and PMNs within periodontal tissues (16). Angipars most probably has reduced gingival LPO and DNA damage because of its main component called coumarin. Coumarin has been found to reduce synthesis of NO in the phagocytes as an anti-inflammatory action (14). On the other hand, since Angipars is suspended in ethanol, thus anti-inflammatory effect of ethanol seems acting in synergism to reduce IL-1β and LPO (17).

The results also indicated that degree of apical migration of JE[the distance between the CEJ and the most coronal portion of JE], the degree of alveolar bone resorption (the distance between the cementoenamel junction (CEJ) and the alveolar bone crest), and PMNs increased in periodontitis group. Angipars caused a significant reduction in apical migration of JE, whereas the amount of alveolar bone resorption decreased insignificantly (p>0.05). The number of PMNs increased significantly in Angipars and vehicle groups compared with periodontitis group. There was no significant difference in the apical migration of JE and alveolar bone resorption between vehicle and periodontitis group. Histological parameters of Angipars group compared with normal group where not significantly different except that there was a significant increase in PMN.

**Figure 1:**
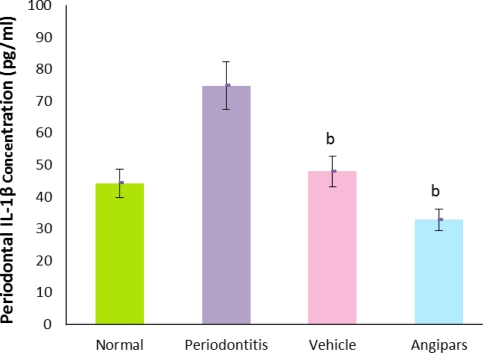
Periodontal IL-1β level Data are mean±SE; IL= interleukin ^a^statistically significant difference with Normal group (p<0.05) ^b^statistically significant difference with Periodontitis group (p<0.05)

**Figure 2:**
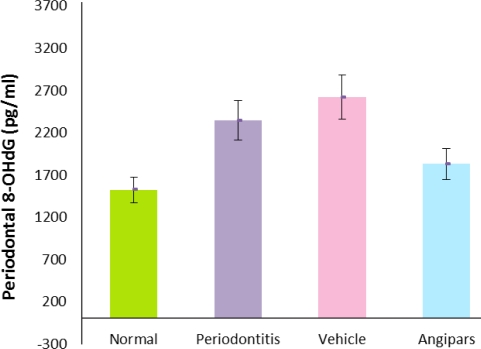
Periodontal 8-OHdG level. Data are mean±SE; 8-OHdG= 8-hydroxydeoxyguanosine ^a^statistically significant difference with Normal group (p<0.05) ^b^statistically significant difference with Periodontitis group (p<0.05)

**Figure 3:**
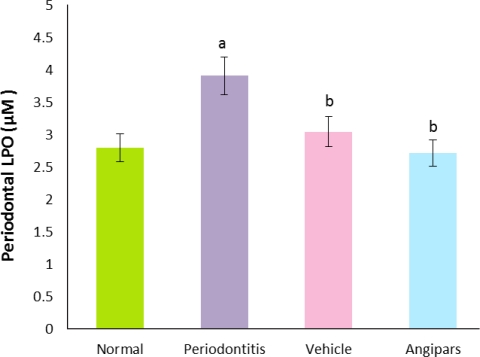
Periodontal LPO level Data are mean±SE; LPO= lipid peroxidation ^a^statistically significant difference with Normal group (p<0.05) ^b^statistically significant difference with Periodontitis group (p<0.05)

**Figure 4:**
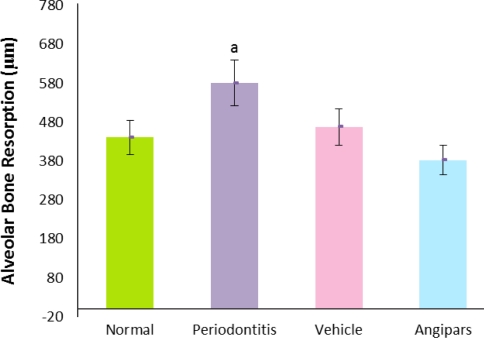
Alveolar bone resorption Data are mean±SE ^a^statistically significant difference with Normal group (p<0.05)

**Figure 5:**
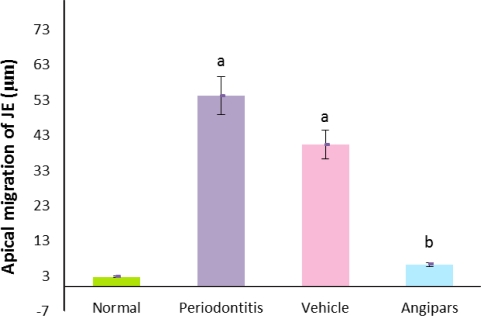
Apical migration of JE Data are mean±SE; JE=Junctional Epithelium ^a^statistically significant difference with Normal group (p<0.05) ^b^statistically significant difference with Periodontitis group (p<0.05)

**Figure 6:**
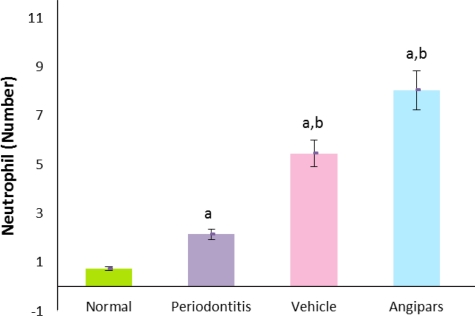
Neutrophil leukocyte infiltration Data are presented as mean±SE ^a^statistically significant difference with Normal group (p<0.05) ^b^statistically significant difference with Periodontitis group (p<0.05)

The alveolar bone resorption in Angipars group was not significantly lower than that of periodontitis group, suggesting that Angipars does not affect the osteoclast activity significantly. Degree of apical migration of JE in Angipars group was significantly lower than that of periodontitis group.

The increase of gingival PMN by Angipars may be explained by its angiogenesis properties that have been reported previously (18). In fact, PMN seem acting more effectively than lymphocytes in protecting microvasculature of periodontitis (19). On the other hand, vehicle group (ethanol) increased PMNs as much as Angipars. Of course, there are conflicting data about angiogenic effects of ethanol that limit us to conclude absolutely. Some studies have shown suppressive effect of ethanol extract of some plants on angiogenesis, whereas others have found inductive properties of ethanol extract of different plants (20, 21). Thus, angiogenic activity of ethanol in periodontitis remains to be elucidated by further studies.

Taking collectively, present findings are adequate to conclude that benefit of Angipars in periodontitis comes from its anti-oxidative properties. This conclusion is supported by a recent clinical trial on diabetic patients which showed beneficial effect ofAngipars on oxidative DNA damage after administration for 3 months (10). Unexpected increase of PMNs by Angipars limits our conclusion but strengthens the hypothesis that chronic inflammatory disorders like periodontitis may requires more time to get best advantage of anti oxidative drugs like Angipars (22). Also regarding role of microbes in pathogenesis of periodontitis, further studies on antimicrobial effects of Angipars are required.
